# Changing Demographics and Prevalence of Body Lice among Homeless Persons, Marseille, France

**DOI:** 10.3201/eid2311.170516

**Published:** 2017-11

**Authors:** Tran Duc Anh Ly, Youssoupha Touré, Clément Calloix, Sékéné Badiaga, Didier Raoult, Hervé Tissot-Dupont, Philippe Brouqui, Philippe Gautret

**Affiliations:** Aix-Marseille-Université, Marseille, France

**Keywords:** Homeless, ectoparasites, body lice, louse, delousing, risk factors, alcohol, alcoholism, homeless, tobacco, smoking, shelter, poverty, hygiene, migrant, France, North Africa, parasites

## Abstract

The prevalence of body lice among 2,288 sheltered homeless persons in the city of Marseille during 2000–2017 was 12.2% and significantly decreased over time. We report a positive association between body lice infestations and older age, duration of stays in France for migrants, frequent consumption of alcohol, and tobacco smoking.

Homeless persons are predisposed to infections because of their poor physical state and lack of hygiene; therefore, outbreaks of contagious diseases are more prevalent among them (*1*,*2*). Body lice infestation prevalence in homeless populations has been shown to be 19.0%–68.0% (*3*–*7*). However, despite the capacity of these ectoparasites to be vectors of several diseases, study of infestation has been minimal among the homeless. To identify potential risk factors for body lice infestation, we analyzed the demographics and chronic medical conditions of the homeless population from Marseille and their variations during 18 years.

## The Study

The protocol for this study was reviewed and approved by the Institutional Review Board and Ethics Committee of Assitance Publique Hôpitaux de Marseille (2010-A01406–33). We conducted cross-sectional, 1-day surveys during 2000–2017 in 2 Marseille shelters (A and B) housing a limit of 300 homeless persons each with a high turnover. Most persons in shelters A and B stay for nights only with no time limitation, but shelter A has a special day/night unit with a 35-bed capacity, dedicated to high-risk sedentary homeless persons whose characteristics include a high level of poverty, poor hygiene, alcoholism, and mental illness. We informed participants of our ongoing study of infectious diseases and that they could receive a complete medical examination free of charge and treatment when needed. Participants volunteered and signed informed consent documents. A medical team interviewed participants by using a standardized questionnaire and physically examined them for the presence of ectoparasites (Technical Appendix). Several delousing measures were administered over time at the 2 shelters (Technical Appendix).

We recruited 2,288 persons, 57% of whom were enrolled in shelter A (Table 1). Most participants were middle-aged men, most of whom were originally from North Africa and settled in France >12 years before the survey was done. A total of 334 (33.2%) migrants reported recurrent travel to their country of origin. Homelessness lasting >1 year accounted for 44.1% of cases.

**Table 1:** Demographics and results of univariate and multivariate analysis of risk factors for body lice infestation among homeless persons, Marseille, France, 2000–2017*

Demographics	Total	Body lice prevalence	Odds ratio (95% CI), p value	
			Univariate analysis	Multivariate analysis
Shelter used				
A†	1,305 (57.0)	90 (10.5)	1.33 (1.01–1.76), 0.041	3.45 (1.37–8.66), 0.008
B	983 (43.0)	152 (13.5)		
Sex				
M	2,161 (95.4)	236 (12.6)		
F	105 (4.6)	5 (5.7)		
Age, y				
Mean (SD)	43.1 (15.0)	NA		
Range	18–86	NA		
<25	221 (10.0)	4 (2.1)		
25–50	1,291 (58.5)	105 (9.5)		
>50	694 (31.5)	121 (19.8)	2.70 (2.04–3.57), <0.0001	2.47 (1.14–5.34), 0.022
Birthplace				
France mainland	597 (26.4)	95 (19.2)	2.18 (1.64–2.89), <0.0001	
France overseas territories	23 (1.0)	6 (30.0)		
North Africa	1,109 (49.0)	95 (9.6)	0.62 (0.47–0.81), <0.0001	
Sub-Saharan Africa	123 (5.4)	14 (13.0)		
Eastern Europe	270 (11.9)	16 (7.0)		
Western Europe	77 (3.4)	10 (14.3)		
Asia	51 (2.3)	3 (6.7)		
Other	11 (0.5)	0		
Duration of residence in France, y				
Mean (SD)	12.2 (16.8)	NA		
Range	0–75	NA		
<1	390 (38.8)	8 (2.3)		
1–5	176 (17.5)	4 (2.6)		
>5‡	438 (43.6)	41 (11.6)	5.46 (2.83–10.55), <0.0001	4.28 (1.79–10.23), 0.001
Visited country of origin since immigration	334 (33.2)	20 (7.4)		
Total no. visits to country of origin since immigration	672 (66.8)	27 (4.8)		
Duration of homelessness, y				
Mean	3.8	NA		
Range	0–57	NA		
<1	1,218 (55.9)	66 (6.3)		
1–5	443 (20.3)	52 (13.4)		
>5	518 (23.8)	112 (24.7)	3.67 (2.77–4.88), <0.0001	

*Values are no. (%) persons except as indicated. Blank cells indicate statistically insignificant results; variables with a prevalence <5.0% were not included in the univariate model. NA, not applicable. †Includes high-risk homeless special unit (33 of the 300 beds in the shelter). ‡North African migrants only: body lice prevalence was 1.6% in persons living in France for <5 y vs. 9.3% in those living in France for >5 y (OR 6.39, 95% CI 2.41–16.95; p<0.0001).

The proportion of France- and Eastern Europe–born homeless persons decreased significantly over time (R^2^ = 0.68 and 0.55, respectively) and the proportion from North Africa increased (R^2^ = 0.64) (Technical Appendix Figure 1). The mean duration of stays in France for migrants and the mean duration of homelessness decreased significantly over time (R^2^ = 0.71 and 0.53, respectively; Technical Appendix Figure 2). 

Among participants, 75% reported smoking tobacco, 60% consuming alcohol, and 20% consuming cannabis (Technical Appendix Figure 2). In addition, 25% of participants had an elevated body mass index; 5.8% were underweight. Tobacco smoking and frequent alcohol consumption decreased significantly during the study period (R^2^ = 0.61 and 0.94, respectively; Technical Appendix Figure 3).

We recorded a high prevalence of pruritus (548 persons [26.9%]), associated with scratch lesions in 306 (17.4%) persons. Overall, 382 (23.3%) participants had lice. The prevalence of differing types of lice was body lice, 12.2% (n = 242); head lice, 4.5% (n = 87); crab lice, 3.2% (n = 53); and scabies, 2.8% (n = 50). The prevalence of body lice decreased significantly during the study period (R^2^ = 0.58) (Technical Appendix Figure 3).

Body lice prevalence was higher in shelter A than in shelter B, increased with age and duration of homelessness, and was higher among persons born in France compared with others (Table 1). Body lice were less common in persons born in North Africa than in those born elsewhere. Body lice prevalence in migrants increased with duration of stay in France. Consuming alcohol frequently, smoking tobacco, and being underweight were associated with an increased risk for body lice (Table 2). In multivariate analyses, only housing in shelter A, older age, duration of stay in France for migrants, frequent consumption of alcohol, and smoking tobacco remained associated with an increased prevalence of body lice. Smoking, alcohol consumption, and underweight prevalence varied according to place of birth (Technical Appendix).

**Table 2:** Results of univariate and multivariate analysis of risk factors for body lice infestation among homeless persons, Marseille, France, 2000–2017*

Risk factor	Total	Body lice prevalence	Odds ratio (95% CI), p value	
			Univariate analysis	Multivariate analysis
Substance use				
Alcohol				
Never	892 (39.8)	29 (3.7)		
Sometimes	623 (27.8)	55 (9.8)		
Frequently	729 (32.5)	155 (25.1)	5.01 (3.77–6.68), <0.0001	3.93 (1.85–8.36), <0.0001
Tobacco				
Never	576 (25.5)	20 (4.0)		
Yes	1680 (74.5)	218 (14.9)	4.16 (2.60–6.65), <0.0001	2.46 (1.04–5.79), 0.04
Cannabis (never)	921 (82.1)	70 (8.1)		
Cannabis	201 (17.9)	23 (11.9)		
Injected substances	19 (1.1)	5 (27.8)		
Nasally inhaled substances	40 (3.0)	7 (24.1)		
Drug substitutes	25 (1.5)	5 (21.7)		
Medical conditions				
COPD	38 (9.9)	11 (5.6)		
Asthma	63 (6.1)	3 (6.5)		
Bronchitis	44 (4.3)	4 (12.1)		
Cancer	6 (0.9)	0		
Diabetes	47 (5.8)	1 (2.2)		
Hepatitis	22 (2.8)	7 (33.3)		
History of pulmonary TB	55 (4.2)	3 (7.1)		
Weight				
Mean BMI (SD)	23.8 (4.2)			
BMI range	13.5–65.0			
Underweight	97 (5.8)	14 (17.9)	2.998 (1.53–5.90), 0.001	
Normal weight	1,009 (60.3)	93 (10.8)		
Overweight	439 (26.2)	23 (6.0)		
Obesity	129 (7.7)	11 (9.6)		
*Only statistically significant results are reported; blank cells indicate statistically insignificant results. Variables with a prevalence <5.0% were not included in the univariate model. ND, not done.				

## Conclusions

In this survey, we observed significant changes over time in the demographic characteristics of the homeless population in Marseille. Overall, France-born, long-term homeless persons were progressively replaced by migrants of North Africa origin, who had a shorter duration of homelessness. Concurrently, the prevalence of frequent alcohol consumption and tobacco smoking decreased over time. France-born homeless persons were more prone to alcoholism and smoking habits, and those originating from North Africa were less likely to be frequent consumers of alcohol.

The decrease over time in overall body lice prevalence could be attributed to the changes in the characteristics of the population and also to the effects of delousing interventions conducted in the shelters. We identified several independent risk factors for body lice, including older age, residence duration in France of migrants, frequent alcohol consumption, and tobacco smoking. The latter 2 factors are likely correlative because they are markers of poor self-care, which may be associated with risk of body lice infestation. 

These results correlate with the observation of very high prevalence of body lice found in a specific survey by our team of 33 high-risk homeless persons from shelter A in which a 84.9% prevalence was found, compared to an estimated 22.0% prevalence in the overall population of shelter A over the same period of time (*8*). Those results corroborate our observation of a higher overall prevalence of body lice in shelter A than in shelter B. The subpopulation of the homeless persons with an elevated level of high-risk behaviors, housed in the special sector of shelter A, may have acted as a source of reinfestation for the other persons in this shelter. Unfortunately, being housed in shelter A’s special unit was not documented on a regular basis in our surveys. 

We found no other published study addressing risk factor analysis for body lice among sheltered homeless persons. In a Paris survey, A. Arnaud et al. conducted a risk factor analysis among homeless persons sleeping in public areas only, and body lice prevalence was associated with a history of pubic lice, begging, and not attending municipal showers (*9*). In a survey of homeless persons in San Francisco who were consulting for possible lice infestation, male gender, African American ethnicity, and sleeping outdoors were significantly associated with having body lice (*7*).

Our observation that the strongest determinant of body lice in the homeless was alcoholism correlates with previous observations that trench fever is associated with a history of alcoholism (*10*–*13*). Because body lice are known vectors of trench fever, which was the most frequently reported vectorborne infection identified in homeless persons in Europe and the US during 1990–2014, targeted removal of lice should specifically reduce its incidence in this population (*14*,*15*). 

Our survey has several limitations. The homeless persons were not randomly selected, so those who had skin disease symptoms might have been more prone to enroll in the survey because a free medical examination was offered. Our results represent only homeless persons provided with shelter and cannot be extrapolated to those sleeping outside, where a higher prevalence of body lice has been reported (*9*). 

Notwithstanding these limitations, these results demonstrate the value of investigating the homeless in shelters directly to estimate the prevalence of body lice and its risk factors. Our survey indicates that demographic factors, addictions, and being underweight are factors associated with body lice risk, which may be used to better target populations for delousing measures. 

Technical Appendix Case definition, statistical analysis details, chronology of delousing measures, and demographic effects on behaviors.
