# Supplementary material for: Changing Demographics and Prevalence of Body Lice among Homeless Persons, Marseille, France
Source: Emerg Infect Dis. 2017 Nov;23(11):1894–7. doi: 10.3201/eid2311.170516 (PMC5652409; doi:10.3201/eid2311.170516)
Supplement: Technical Appendix — Case definition, statistical analysis details, chronology of delousing measures, and demographic effects on behaviors. [file 17-0516-Techapp-s1.pdf]

Article DOI: https://doi.org/10.3201/eid2311.170516

Changing Demographics and Prevalence of Body Lice among Homeless Persons, Marseille, France

Technical Appendix

Case Definition for Ectoparasite Infestation

Medical doctors conducted clinical examination of hair, body, and clothing. Lice in the clothing or on the body below the neck were considered a positive indication of body lice infestation. Any lice found on the head with the presence of nits were considered to be head lice. Finding lice on pubic hairs was considered a positive indication for crab lice infestation. Lice were collected from all persons using forceps and identified at the species level (*Pediculus* or *Phtirius*) by a medical entomologist based on morphological criteria. Distinction between *P. humanus humanus* (body lice) and *P. humanus capitis* (head lice) was based on recovery sites (clothing and body versus scalp and nape).

A case of scabies was defined on clinical criteria by trained physicians. Individuals were considered affected by scabies when presenting with itchy papular rash with or without vesicles and/or itchy skin-colored scabious burrows in at least two of the following parts of the body: finger webs, flexor surface of the wrist, elbows, armpit, buttocks and genitalia, and breasts.

Statistical Analysis

Statistical analysis was conducted using SPSS (version 22.0). We hypothesized that several factors may influence the outcome of body lice prevalence, including demographics and chronic medical conditions. The Pearson's Chi-square test and Fisher exact test, when appropriate, were applied to analyze the categorical variables. P values of ≥0.05 were considered significant. Variation over time was assessed statistically as the proportion of variance explained by year and considered adequately fitted if the coefficient of determination [R^2 statistic] was >50%.

Chronology of Delousing Measures Conducted at Marseille Shelters

A general practitioner is available for medical care 2 or 3 times per week and a registered nurse is available daily in each shelter to provide medical care. Since 2001, individuals presenting with lice have been provided showers, new clothes, and laundry. Given that lice were recovered not only from the homeless' clothes but also from the bedding, blankets were either treated with permethrin or boiled regularly (*1*). These measures, however, have failed to control body-louse infestation in these shelters. In addition, from 2004 to 2005, 3 doses of oral ivermectin (12 mg each), administered at 7-day intervals where provided to patients having lice, resulting in a dramatic reduction of lice at day 14 (from 84.5% infestation rate at day 0 to 18.5% at day 14), but was followed by a recrudescence of lice infestation at day 45 (60.7%) (*2*). Since 2006, a single dose regimen of 24 mg ivermectin has been used and proved effective at day 14, reducing the prevalence of pruritus (from 92.7% at day 0 to 68.6% at day 14) (*3*). This effect was however not sustained at day 28 (79.2% pruritus), very likely because of reinfestation from other untreated subjects in the shelters (*3*). In 2011, body lice–infested homeless persons were randomly assigned to receive 0.4% permethrin–impregnated underwear or an identical-appearing placebo for 45 days. Permethrin–impregnated underwear was more efficient than placebo at eliminating body louse infestations by day 14 (from 100% infestation rates at day 0 to 72% at day 14 in the permethrin group, compared to 91% in the placebo group). However, this difference was not sustained on day 45 with the prevalence of permethrin-resistant lice significantly more frequent in the permethrin group than in the placebo group. This strategy was discontinued in 2012 (*4*). The shelter's staff was informed of the results of the studies every year following each cross-sectional survey or delousing trials and became aware of recommendations for delousing. The current strategy applied in the two shelters is to offer systematic empirical 24 mg single dose ivermectin to treat pruritus in individuals seeking help in association with washing and changes of clothing and linens and treatment of blankets with insecticides. Since the effect of ivermectin is transient in this context, repeated use is recommended, given that the drug is remarkably safe (*4*).

Effect of Place of Birth on Smoking, Alcohol Consumption, and Underweight Prevalence

Frequent consumption of alcohol prevalence was of 47.5% in individuals born in France, compared to 27.1% in others (Odds ratio = 2.43 [2.00–2.95], $p<0.0001$) and 23.2% in those born in North Africa compared to 41.6% in others (Odds ratio = 0.42 [0.35–0.50], $p<0.0001$). Smoking prevalence was of 84.5% in French-born individuals compared to 70.8% in others (Odds ratio = 2.24 [1.75–2.86], $p = 0.0001$) and 70.4% in those born in North Africa compared to 78.4% in others (Odds

ratio = 0.65 [0.54–0.79], p<0.0001). 9.3% of French-born individuals were underweight compared to 4.7% of others (Odds ratio = 2.08 [1.36–3.19], p = 0.001) and 5.1% of individuals originating from North Africa were underweight compared to 6.6% of others (Odds ratio = 0.76 [0.50–1.14], p = 0.18).

References

1. Brouqui P, Stein A, Dupont HT, Gallian P, Badiaga S, Rolain JM, et al. Ectoparasitism and vector-borne diseases in 930 homeless people from Marseilles. Medicine (Baltimore). 2005;84:61–8. PubMed http://dx.doi.org/10.1097/01.md.0000152373.07500.6e

2. Foucault C, Ranque S, Badiaga S, Rovery C, Raoult D, Brouqui P. Oral ivermectin in the treatment of body lice. J Infect Dis. 2006;193:474–6. PubMed http://dx.doi.org/10.1086/499279

3. Badiaga S, Foucault C, Rogier C, Doudier B, Rovery C, Dupont HT, et al. The effect of a single dose of oral ivermectin on pruritus in the homeless. J Antimicrob Chemother. 2008;62:404–9. PubMed http://dx.doi.org/10.1093/jac/dkn161

4. Benkouiten S, Drali R, Badiaga S, Veracx A, Giorgi R, Raoult D, et al. Effect of permethrin-impregnated underwear on body lice in sheltered homeless persons: a randomized controlled trial. JAMA Dermatol. 2014;150:273–9. PubMed http://dx.doi.org/10.1001/jamadermatol.2013.6398

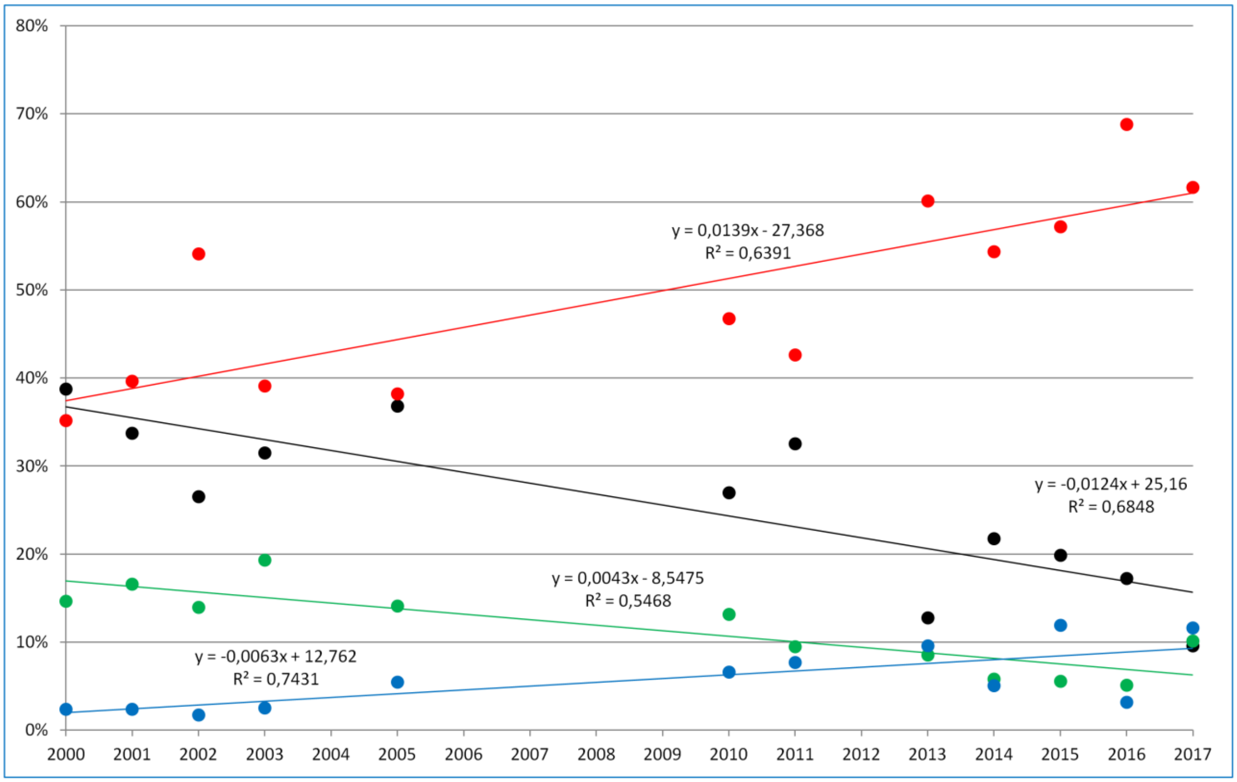

Technical Appendix Figure 1. Birthplace (%) according to year in study of risk factors for body lice infestation among homeless persons in Marseille, France, 2000–2017. Black dots: France; red dots: North Africa; green dots: Eastern Europe; blue dots: sub-Saharan Africa.

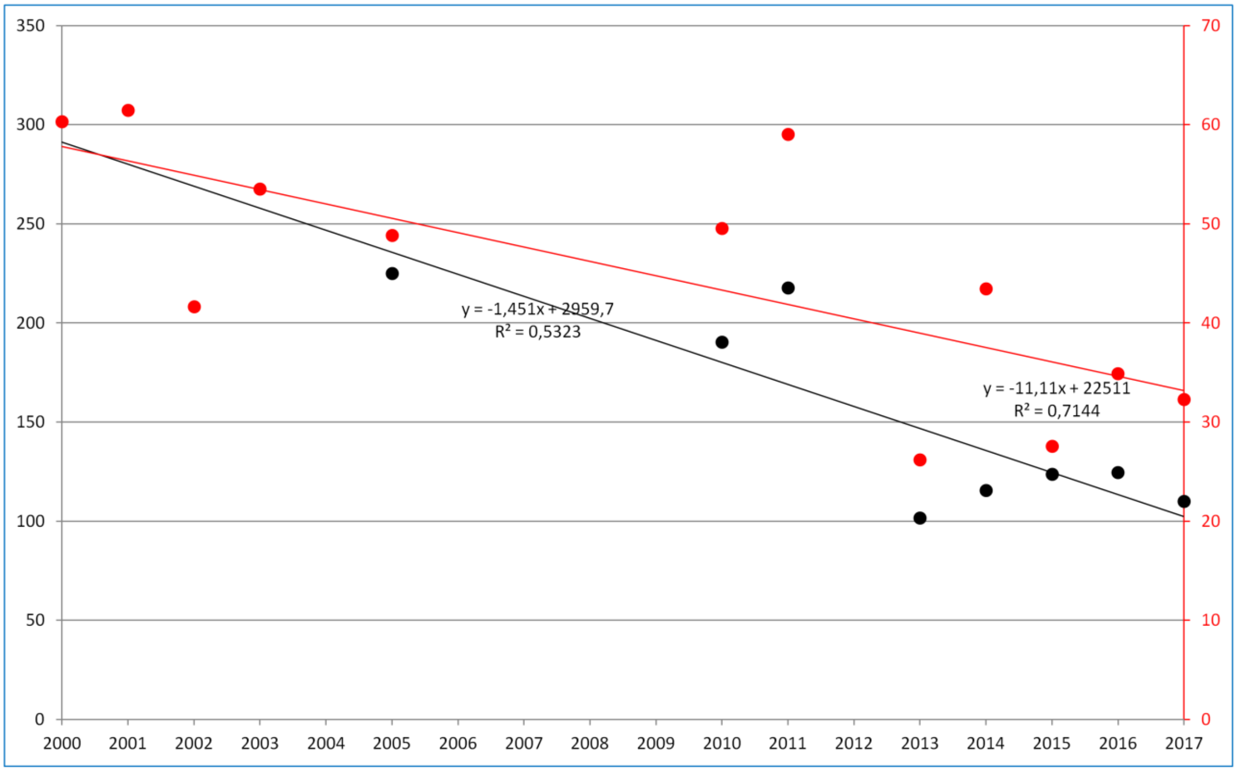

Technical Appendix Figure 2. Mean duration of residence in France, in months, for migrants (black dots); and mean duration of homelessness in months (red dots) according to year in study of risk factors for body lice infestation among homeless persons in Marseille, France, 2000–2017.

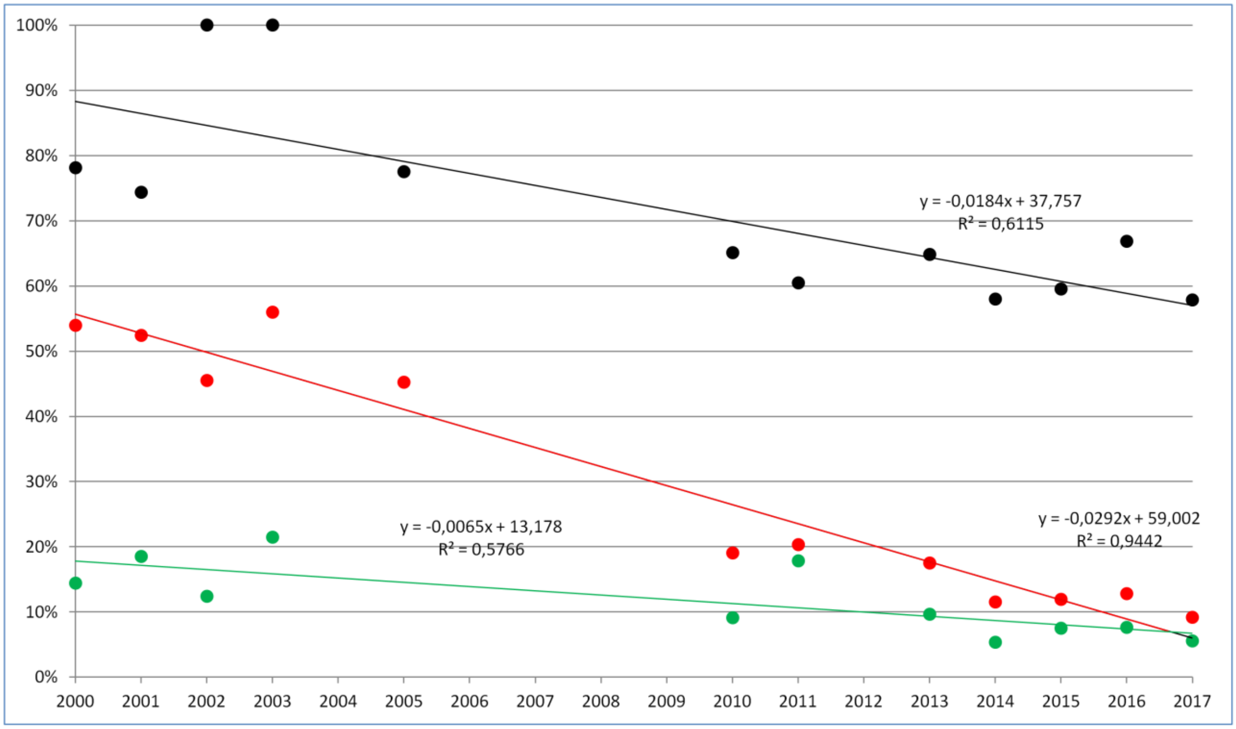

Technical Appendix Figure 3. Prevalence (%) of smoking habits (black dots), frequent alcohol consumption (red dots) and body lice infestation (green dots) according to year in study of risk factors for body lice infestation among homeless persons in Marseille, France, 2000–2017.
